# Emotional Dysregulation as a Clinically Relevant Dimension of Adult ADHD: A Multidimensional Clinical Study

**DOI:** 10.3390/brainsci16060577

**Published:** 2026-05-29

**Authors:** Paola Landi, Miriam Olivola, Arianna De Ciechi, Luca Giacovelli, Yacob Levin Reibman, Viviana Venturi, Vera Viganò, Bernardo Dell’Osso

**Affiliations:** 1 Department of Mental Health and Addiction Services, Azienda Socio Sanitaria Territoriale (ASST) Fatebenefratelli-Sacco, 20100 Milan, Italy; miriam.olivola@asst-fbf-sacco.it (M.O.); giacovelli.luca@asst-fbf-sacco.it (L.G.);; 2 Department of Brain and Behavioral Sciences, University of Pavia, 27100 Pavia, Italy; 3 Department of Mental Health, University of Milan, 20100 Milan, Italy; 4 Department of Philosophy, Social Sciences and Education, University of Perugia, 06100 Perugia, Italy; arianna.deciechi@dottorandi.unipg.it; 5 Department of Biomedical and Clinical Sciences “Luigi Sacco”, University of Milan, 20157 Milan, Italy; 6 “Aldo Ravelli” Center for Nanotechnology and Neurostimulation, University of Milan, 20157 Milan, Italy; 7 Department of Psychiatry and Behavioral Sciences, Stanford University, Stanford, CA 94305, USA; 8 Centro per lo Studio dei Meccanismi Molecolari alla Base delle Patologie Neuro-Psico-Geriatriche, University of Milan, 20100 Milan, Italy

**Keywords:** ADHD, emotional dysregulation, DERS, substance use disorder, impulsivity, internet addiction, psychiatric comorbidity, adult

## Abstract

**Highlights:**

**What are the main findings?**

- Adults with ADHD showed markedly elevated emotional dysregulation (DERS total M = 115.02).
- Combined ADHD presentation was associated with significantly higher dysregulation than inattentive presentation.
- Current SUD, current ADHD symptoms, retrospective childhood ADHD symptoms, trait anxiety, and ADHD-related quality of life were independently associated with DERS total score.
- Impulsivity showed only a modest association with emotional dysregulation and was not an independent predictor in multivariate models.

**What are the implications of the main findings?**

- Findings highlight the clinical relevance of assessing emotion regulation difficulties in adults with ADHD, particularly in relation to comorbidity, substance use, symptom burden, and quality of life.

**Abstract:**

Background: Emotional dysregulation (ED) is increasingly recognized as a clinically relevant dimension of adult attention-deficit/hyperactivity disorder (ADHD), yet its multidimensional structure and independent contribution beyond impulsivity and psychiatric comorbidity remain incompletely understood. This study aimed to characterize the profile of ED and its clinical correlates in a large, well-characterized clinical sample of adults with ADHD. Methods: In this cross-sectional observational study, 231 adults with a DSM-5 diagnosis of ADHD, confirmed through structured interview (DIVA 5), completed the Difficulties in Emotion Regulation Scale (DERS). Associations between ED and ADHD presentation, psychiatric comorbidity, current substance use disorder (SUD), impulsivity (BIS-11), problematic internet use (IAT), camouflaging behaviors (CAT-Q), and clinical functioning were examined using independent-samples *t*-tests, Pearson correlation analyses, and multiple linear regression models. Results: Participants showed elevated emotional dysregulation relative to reference data. When DERS subscales were interpreted against Italian reference values, the largest elevation was observed for Clarity, followed by Goals, Nonacceptance, and Impulse, whereas Strategies showed a more modest elevation and Awareness was only slightly higher than the reference value. The combined ADHD presentation was associated with significantly greater dysregulation compared to the predominantly inattentive presentation. Higher DERS total scores were observed among participants with combined ADHD presentation, psychiatric comorbidity, and current SUD. DERS total score was positively correlated with current ADHD symptoms, retrospective childhood ADHD symptoms, depressive symptoms, state and trait anxiety, impulsivity, problematic internet use, and camouflaging behaviors, and negatively correlated with ADHD-related quality of life. In multiple regression models, current SUD, current ADHD symptoms, retrospective childhood ADHD symptoms, trait anxiety, and ADHD-related quality of life were independently associated with DERS total score. Conclusions: Emotional dysregulation in adult ADHD represents a clinically relevant affective dimension that is only partially accounted for by impulsivity and is closely associated with psychosocial impairment and maladaptive coping behaviors. These findings support an integrated affective–executive framework for the assessment and treatment of adult ADHD, with implications for targeted, skills-based interventions addressing emotional regulation across clinical subgroups.

## 1. Introduction

Attention-Deficit/Hyperactivity Disorder (ADHD) is a neurodevelopmental condition characterized by persistent patterns of inattention, hyperactivity, and impulsivity that impair global functioning and development [1]. Although traditionally conceptualized as a childhood disorder, ADHD frequently persists into adulthood, with prevalence estimates ranging from 2.5% to 4.4% in adult populations [2,3]. Adult ADHD is associated with substantial functional impairment across multiple life domains, including academic achievement, occupational performance, interpersonal relationships, and overall quality of life [4,5].

The clinical presentation of ADHD in adults often differs from childhood manifestations; while symptoms of overt hyperactivity typically diminish, executive dysfunction and emotional regulation difficulties become more prominent [6]. Furthermore, adults with ADHD often face significant delays in diagnosis and treatment initiation, leading to prolonged periods of untreated illness—defined here as the interval between symptom onset and first therapeutic contact—and the cumulative accrual of functional impairments [7]. Diagnostic delay, defined as the time elapsed between first psychiatric contact and formal ADHD diagnosis, further compounds this burden. The disorder also exhibits high rates of comorbidity with other psychiatric conditions including mood, anxiety and substance use which further complicate the clinical landscape and treatment strategies [2].

Emotional dysregulation (ED)—defined as difficulty in modulating emotional experiences and expressions in contextually appropriate ways [8]—has emerged as a clinically significant feature of ADHD that extends beyond the core diagnostic criteria [9]. Individuals with ADHD frequently report impaired emotional control, heightened emotional reactivity, rapid mood shifts, and challenges in managing frustration or anger. These emotional deficits contribute substantially to functional impairment and may persist even when core ADHD symptoms are effectively managed with pharmacotherapy [10].

The relationship between ADHD and emotional dysregulation is complex and multifaceted. Neurobiological research suggests that both ADHD and ED share overlapping neural substrates, particularly within the prefrontal–limbic circuits involved in emotion regulation and executive control [9,11]. Recent studies on dynamic neural network interactions have further suggested that compensatory pathways in altered neurological states may modulate the clinical expression of regulatory deficits, highlighting the necessity of an integrated affective–executive framework. In this context, ED in ADHD may arise from deficits in executive functions that typically support emotional modulation, such as working memory, inhibitory control, and cognitive flexibility [12]. Additionally, the chronic experience of failure, social rejection, and frustration associated with untreated ADHD may further catalyze the development of maladaptive emotional patterns [13,14].

The clinical significance of emotional dysregulation in ADHD is substantial. Emotional difficulties are related to poorer treatment response, an increased risk of comorbid psychiatric disorders, higher rates of substance misuse, impaired socio-occupational functioning, and reduced quality of life [10,15]. Despite its clinical impact, ED remains excluded from current formal diagnostic criteria for ADHD, and systematic assessments of emotional functioning are not yet routinely integrated into standard diagnostic or treatment protocols [16].

The Difficulties in Emotion Regulation Scale (DERS) is a comprehensive, multidimensional self-report instrument designed to assess clinically relevant challenges in emotion regulation [8]. Developed by Gratz and Roemer, the DERS conceptualizes emotion regulation as a multifaceted construct that encompasses the awareness, understanding, and acceptance of emotions; the ability to maintain impulse control and goal-directed behavior when experiencing negative affect; and the access to effective modulation strategies.

The DERS has demonstrated robust psychometric properties across diverse populations and is widely utilized in research examining emotion regulation across various psychiatric disorders, including ADHD [17,18]. Its multidimensional structure facilitates a nuanced assessment of specific regulatory deficits, which may, in turn, inform targeted therapeutic interventions.

Elucidating the nature and correlates of emotional dysregulation in adults with ADHD carries significant clinical implications. First, the systematic assessment of emotional functioning could enhance diagnostic accuracy and refine treatment planning [7]. Second, identifying specific emotion regulation deficits may guide the selection of tailored psychotherapeutic interventions, such as Dialectical Behavior Therapy (DBT) or specialized emotion regulation skills training [19,20]. Third, understanding the interplay between ED and clinical variables such as substance use, psychiatric comorbidity, and functional impairment can inform risk stratification and decisions regarding treatment intensity [10,21].

Despite the growing recognition of emotional dysregulation in ADHD, several critical questions remain. The relationship between ED and other core ADHD features, particularly impulsivity, requires further clarification [22]. Furthermore, the association between ED and emerging constructs, such as “camouflaging” behaviors (strategies used to mask or compensate for neurodevelopmental differences), has not been systematically investigated in ADHD populations. Additionally, the clinical and demographic predictors of severe emotional dysregulation in ADHD remain incompletely characterized.

In the present study, we examine emotional dysregulation as a clinically relevant affective-regulatory dimension in adults with ADHD, while acknowledging that its diagnostic status cannot be determined from a cross-sectional clinical sample without a non-ADHD comparison group.

Beyond ADHD-specific mechanisms, emerging evidence from broader neurological research suggests that emotional and cognitive dysregulation may reflect shared principles of neural vulnerability and compensation across conditions. For instance, studies in progressive neurological disorders have highlighted the role of dynamic compensatory networks and molecular processes such as alternative splicing and neuroinflammatory pathways in shaping adaptive and maladaptive brain responses to stressors and dysfunction. These mechanisms may contribute to the heterogeneity of clinical phenotypes and the variability in resilience observed across individuals.

Although direct evidence in ADHD remains limited, situating emotional dysregulation within this broader neurobiological framework may help conceptualize it as part of a continuum of adaptive and maladaptive neural responses, rather than as an isolated symptom domain.

### Study Objectives

The primary objective was to characterize emotional dysregulation in adults with ADHD using the DERS, exploring its associations with impulsivity (BIS-11), ADHD symptom severity (DIVA 5), camouflaging behaviors (CAT-Q), and problematic internet use (IAT). Secondary objectives involved examining differences across clinical subgroups including psychiatric comorbidity, substance use disorder, and academic functioning and identifying predictors of heightened emotional dysregulation.

## 2. Materials and Methods

### 2.1. Study Design and Setting

This cross-sectional observational study was conducted at the specialized Adult ADHD Center of ASST Fatebenefratelli-Sacco in Milan, Italy. This tier-2 outpatient mental health service is dedicated to the comprehensive assessment and treatment of adults with ADHD and related comorbidities, providing diagnostic evaluations, pharmacological management, and psychosocial interventions. The service specifically caters to a population aged 18–35 years, an age range established by its integration into regional clinical projects for young adults.

Data were prospectively collected from consecutive adult patients evaluated at the service between January 2020 and January 2026. Adults diagnosed with ADHD according to DSM-5 criteria through a structured clinical assessment—including the Diagnostic Interview for ADHD in Adults (DIVA 5)—were invited to participate.

A total of 231 individuals met the DSM-5 diagnostic criteria for ADHD and were included in the study sample. Descriptive socio-demographic and clinical characteristics are reported for the total sample. Due to missing data on certain self-report measures (ranging from 0.4% to 9.1% across variables), the effective sample size varied slightly by analysis. Complete-case analyses were conducted for each model, resulting in analytic samples ranging from N = 210 to *N* = 212 for the primary correlational and regression analyses.

### 2.2. Participants

#### 2.2.1. Inclusion Criteria

Participants were eligible for inclusion if they:

1. Were aged between 18 and 35 years;
2. Met DSM-5 diagnostic criteria for ADHD based on a comprehensive clinical assessment, including the DIVA 5;
3. Were able to provide written informed consent;
4. Demonstrated sufficient proficiency in the Italian language to reliably complete self-report questionnaires.

#### 2.2.2. Exclusion Criteria

Participants were excluded if they:

1. Presented with active psychotic symptoms or severe cognitive impairment that could compromise the validity of self-report measures;
2. Declined participation in the study protocol;
3. Had missing or incomplete data regarding primary outcome variables;
4. Had a comorbid personality disorder.

#### 2.2.3. Sample Characteristics—See Table 1

Socio-demographic and clinical characteristics of the sample are reported in Table 1. The sample included 231 adults with ADHD. The mean age was 26.05 years (*SD* = 4.92), and most participants were male. Psychiatric comorbidity was present in 74.0% of the sample, and current substance use disorder was reported by 25.1% of participants. Academic difficulties were highly prevalent, with 92.6% of participants reporting school difficulties, 62.3% reporting grade repetition, and 35.1% reporting study interruption.

**Table 1:** Socio-Demographic and Clinical Characteristics of the Sample (N = 231).

Variable	M (SD) or n (%)
Continuous variables	
Age, years	26.05 (4.92)
Age at ADHD diagnosis	23.72 (6.80)
Age at first psychiatric contact	20.50 (7.26)
Age at first ADHD treatment	24.51 (6.27)
Duration of untreated illness (DUI), years	11.98 (6.67)
Diagnostic delay, years	3.48 (5.57)
Gender	
Male	136 (58.9)
Female	94 (41.1)
Did not disclose	1 (0.4)
Educational level	
Primary school	1 (0.4)
Middle school	34 (14.7)
High school	125 (54.1)
University degree	69 (29.9)
Did not disclose	2 (0.9)
Employment status	
Unemployed	41 (17.7)
Temporary/part-time employment	62 (26.8)
Permanent employment	54 (23.4)
Other	72 (31.2)
Did not disclose	2 (0.9)
Marital status	
Single	176 (76.2)
Married/cohabiting	52 (22.5)
Separated	1 (0.4)
Did not disclose	2 (0.9)
Clinical comorbidity	
Psychiatric comorbidity, yes	171 (74.0)
Neurodevelopmental comorbidity, yes	73 (31.6)
Organic comorbidity, yes	68 (29.4)
Past substance use disorder, yes	82 (35.5)
Current substance use disorder, yes	58 (25.1)
Clinical severity indicators	
Lifetime psychiatric hospitalizations, yes	34 (14.7)
Lifetime suicide attempts, yes	26 (11.3)
Academic history	
Study interruption, yes	81 (35.1)
Grade repetition, yes	144 (62.3)
School difficulties, yes	214 (92.6)
Family history	
Family history of ADHD, yes	40 (17.3)
Family history of psychiatric disorders, yes	126 (54.5)
ADHD treatment	
Previous ADHD pharmacotherapy, yes	87 (37.7)
Current ADHD pharmacotherapy: atomoxetine	83 (35.9)
Current ADHD pharmacotherapy: methylphenidate	104 (45.0)
Current ADHD pharmacotherapy: bupropion	20 (8.7)
Current ADHD pharmacotherapy: other	16 (6.9)
No current pharmacotherapy	6 (2.6)
Did not disclose	2 (0.9)
Current psychotherapy, yes	97 (42.0)
Type of suicide attempt among participants with lifetime suicide attempts (n = 26)	
Medication ingestion/overdose	9 (34.6)
Hanging	2 (7.7)
Self-cutting	3 (11.5)
Agitated self-injury	5 (19.2)
Other/unspecified	6 (23.1)
Did not disclose	1 (3.8)

Note. DUI = duration of untreated illness. DUI was defined as the number of years between ADHD symptom onset and first therapeutic contact. Diagnostic delay was defined as the number of years between first psychiatric contact and ADHD diagnosis.

### 2.3. Procedures

#### Diagnostic Assessment

Participants underwent a comprehensive diagnostic evaluation conducted by expert clinicians specializing in adult ADHD. The diagnostic protocol comprised:

1. Detailed clinical history taking via semi-structured patient interviews;
2. Administration of screening instruments for both adult and childhood symptoms (ASRS and WURS);
3. Diagnostic confirmation using the DIVA 5 interview, administered to both the patient and a family member to corroborate the clinical diagnosis through informant report;
4. Assessment of functional impairment and quality of life (AAQoL);
5. Evaluation of psychiatric comorbidities through standardized instruments (ZUNG, STAI, SCID-II, MDQ);
6. Psychometric assessment of behavioral traits frequently associated with ADHD, including problematic internet use (IAT), camouflaging behaviors (CAT-Q), impulsivity (BIS-11), and emotional dysregulation (DERS);
7. Review of all available collateral information.

The final ADHD diagnosis was established by consensus within the multidisciplinary clinical team.

Data were prospectively gathered during the initial evaluation or throughout the early phases of treatment. All findings were recorded in a secure electronic database, adhering to stringent safeguards to ensure participant confidentiality and data protection.

### 2.4. Assessment Instruments

#### 2.4.1. Adult ADHD Self-Report Scale (ASRS)

The ASRS is an 18-item self-report scale based on DSM-IV criteria for ADHD [23,24]. It assesses the frequency of recent ADHD symptoms in adults, with items rated on a 5-point frequency scale. The ASRS has demonstrated good sensitivity and specificity for ADHD diagnosis and is widely used to assess symptom severity in clinical and research settings.

#### 2.4.2. Wender Utah Rating Scale (WURS)

This is a scale used in the diagnosis of attention deficit hyperactivity disorder based on behavior and feelings experienced during childhood [25]. The self-report questionnaire administered to the patient consists of 61 items, 25 of which are highly relevant items for ADHD and can help link childhood symptoms with patterns of behavior in adulthood. Each of the questions in the retrospective scale has 4 answer choices, awarded points from 0 to 4.

#### 2.4.3. Diagnostic Interview for ADHD in Adults (DIVA 5.0)

DIVA-5, the structured diagnostic interview for ADHD in adults. This scale was developed by Kooij and colleagues and is based on the DSM-5 criteria for ADHD [26]. The interview focuses exclusively on the key symptoms of ADHD and does not assess any co-occurring psychiatric disorders. Collateral information from family members, partners, or available clinical records is used to corroborate the patient’s report. The DIVA is divided into three parts, each of which considers both childhood/adolescence and adulthood and includes criteria related to Attention (A1), Hyperactivity/Impulsivity (A2) and the age of onset of ADHD-related symptoms [27].

#### 2.4.4. Zung Depression Rating Scale (Zung—ZDRS)

The ZDRS is a self-administered questionnaire used to assess a patient’s depressive state using 20 items that investigate the four common characteristics of depression: pervasiveness, somatic symptoms, psychomotor symptoms and other comorbid disorders [28]. The total score ranges from 20 to 80. The cut-off recommended by Zung was an index score of 50 or higher [29,30].

#### 2.4.5. State Trait Anxiety Inventory (STAI-Y)

The STAI-Y [31] is the most widely used self-report questionnaire for assessing anxiety in research and clinical practice [32]. There are two subscales within this measure. First, the State Anxiety Scale (S-Anxiety or STAI-Y1) assesses the current state of anxiety. The Trait Anxiety Scale (T-Anxiety or STAI-Y2) assesses relatively stable aspects of ‘anxiety predisposition’. The STAI consists of 40 items rated on a Likert scale. The score range for each subtest is 20 to 80, with higher scores indicating greater anxiety.

#### 2.4.6. Mood Disorder Questionnaire (MDQ)

The MDQ is a self-report instrument that analyses bipolar I and II disorders using 13 items (yes/no) derived from both the DSM-IV criteria and clinical experience [33]. A positive screening of the criteria requires that the patient has answered positively more than 7 times in the first 13 questions, or that they have answered YES to question number 14 or 15, or that all three of the above conditions have been met [34].

#### 2.4.7. Structured Clinical Interview for DSM-5 (SCID-II and SCID-5-PD)

The SCID-II and SCID-5-PD (Personality Disorder) are semi-structured clinical interviews consisting of 90 items that investigate the categories of avoidant, dependent, obsessive–compulsive, paranoid, schizotypal, schizoid, histrionic, narcissistic, borderline and antisocial personality disorders, as well as the criteria for conduct disorder [35].

#### 2.4.8. Difficulties in Emotion Regulation Scale (DERS)

The DERS is a 36-item self-report questionnaire that assesses multiple dimensions of emotion regulation difficulties [8]. Items are rated on a 5-point Likert scale. The DERS yields a total score and six subscale scores. The subscales are Nonacceptance of Emotional Responses (nonacceptance), Difficulty Engaging in Goal-Directed Behavior (Goals), Impulse Control Difficulties (Impulse), Lack of Emotional Awareness (Awareness), Limited Access to Emotion Regulation Strategies (Strategies), Lack of Emotional Clarity (Clarity).

Higher scores indicate greater emotion regulation difficulties [36,37].

#### 2.4.9. Barratt Impulsiveness Scale-11 (BIS-11)

The BIS-11 is a 30-item self-report questionnaire that assesses impulsivity across three dimensions: Attentional Impulsiveness, Motor Impulsiveness, and Non-planning Impulsiveness [38]. Items are rated on a 4-point scale [39].

#### 2.4.10. Camouflaging Autistic Traits Questionnaire (CAT-Q)

The CAT-Q is a 25-item self-report measure designed to assess camouflaging behaviors which are strategies used to mask or compensate for social-communication difficulties [40]. The measure yields a total score and three subscale scores: Compensation, Masking, and Assimilation. While originally developed for autism spectrum conditions, camouflaging behaviors have been observed in other neurodevelopmental conditions, including ADHD [41]. Items are rated on a 7-point Likert scale.

#### 2.4.11. Internet Addiction Test (IAT)

The IAT is a 20-item self-report measure that assesses problematic internet use and internet addiction [42]. Items assess the extent to which internet use affects daily routine, social life, productivity, sleeping patterns, and feelings. Items are rated on a 5-point frequency scale.

### 2.5. Socio-Demographic and Clinical Variables

Comprehensive socio-demographic and clinical data were collected through a structured interview, including: age, gender, educational level, employment status, marital status, age at ADHD diagnosis, age at symptom onset, duration of untreated illness, academic history (study interruption, grade repetition, school difficulties), psychiatric comorbidity, neurodevelopmental comorbidity, organic comorbidity, substance use disorder history (past and current), family psychiatric history, ADHD treatment history (pharmacotherapy and psychotherapy), and clinical severity indicators (hospitalizations, suicide attempts). Bipolar disorder was categorized separately from depressive disorders because of its distinct phenomenology, course, and neurobiological profile relative to major depressive disorder. This decision was made a priori to avoid conflating affectively distinct conditions within a single diagnostic category.

### 2.6. Statistical Analyses

All statistical analyses were conducted using IBM SPSS Statistics, version 27.0 (IBM Corp., Armonk, NY, USA). Statistical significance was set at two-tailed *p* < 0.05. Given the exploratory nature of the study, results were interpreted by considering both statistical significance and effect sizes.

Descriptive statistics were first computed to characterize the sample. Continuous variables were summarized using means and standard deviations, whereas categorical variables were reported as frequencies and percentages. Descriptive statistics were also calculated for the Difficulties in Emotion Regulation Scale (DERS) total score and subscale scores. To facilitate interpretation, DERS scores were presented alongside reference values from the Italian validation study and from the original validation study.

Univariate analyses were then conducted to examine differences in emotional dysregulation across binary socio-demographic and clinical variables. Independent-samples *t*-tests were used to compare DERS total and subscale scores according to sex, ADHD presentation, psychiatric comorbidity, and current substance use disorder. ADHD presentation was analyzed by comparing the combined and inattentive presentations. Participants with a hyperactive/impulsive presentation were excluded from inferential analyses involving ADHD presentation because of the very small subgroup size (*n* = 4), which would have produced unstable estimates. Welch’s correction was applied when Levene’s test indicated unequal variances. For each comparison, means, standard deviations, *t* values, *p* values, and Cohen’s d effect sizes were reported.

Pearson correlation analyses were conducted to examine associations between DERS total and subscale scores and continuous clinical variables, including age, ADHD symptom measures, depressive symptoms, anxiety symptoms, quality of life, impulsivity, problematic internet use, and camouflaging behaviors. Correlation coefficients were interpreted according to conventional thresholds, with *r* = 0.10 considered small, *r* = 0.30 medium, and *r* = 0.50 large.

Finally, variables significantly associated with DERS total score in univariate analyses were entered into multiple linear regression models to examine their independent associations with emotional dysregulation. DERS total score was used as the dependent variable. A simultaneous-entry regression model was first estimated, followed by a stepwise regression procedure to identify the most parsimonious set of variables associated with DERS total score. Regression results were reported using unstandardized coefficients (*B*), standard errors, standardized coefficients (β), *t* values, and *p* values. Missing data were handled using available-case analyses for descriptive, group comparison, and correlational analyses, and listwise deletion for regression models. The results of the assumption-testing analyses (normality, homoscedasticity) are reported in Supplementary Figure S1.

#### 2.6.1. Missing Data

Missing data ranged from 0.4% to 9.1% across variables. Participants with complete data on primary variables (N = 210–212 depending on analysis) were included in correlation and regression analyses. For group comparisons, all available data were used. Missing data patterns were examined using Little’s MCAR test; results indicated that data were missing completely at random (χ^2^ = 187.34, df = 203, *p* = 0.789), supporting the validity of complete-case analysis. Given the low proportion of missing data and MCAR pattern, complete-case analysis was deemed appropriate.

#### 2.6.2. Sensitivity Analyses

To evaluate the robustness of findings, sensitivity analyses were conducted by: (1) repeating primary analyses with and without potential outliers (defined as values > 3 SD from the mean); (2) comparing results using parametric and non-parametric tests where appropriate; and (3) examining whether findings differed when controlling for current ADHD medication status. Results of sensitivity analyses are reported when they differ substantively from primary analyses.

### 2.7. Ethical Considerations

This study was conducted in accordance with the Declaration of Helsinki. All participants provided written informed consent after receiving a complete description of the study. Participants were informed that their participation was voluntary and that declining to participate would not affect their clinical care. All data were de-identified and stored securely to protect participant confidentiality.

## 3. Results

### 3.1. Descriptive Statistics of DERS Scores

Descriptive statistics for the DERS total score and subscales are reported in Table 2. Because DERS subscales differ in score range and reference distributions, subscale scores were interpreted primarily in relation to Italian reference values rather than by direct comparison of raw means. Relative to the Italian reference sample, the largest elevation was observed for Clarity, followed by Goals, Nonacceptance, and Impulse. Strategies showed a more modest elevation, whereas Awareness was only slightly higher than the Italian reference value.

### 3.2. Group Comparisons of DERS Scores

Independent-samples *t*-tests were conducted to compare DERS total and subscale scores across binary socio-demographic and clinical groups. Results are reported in Table 3.

No significant differences emerged between males and females for DERS total score or any subscale. ADHD presentation was significantly associated with DERS total score, with higher scores in the combined presentation group than in the inattentive presentation group. At the subscale level, significant differences were observed for Nonacceptance, Strategies, and Awareness. Psychiatric comorbidity was associated with higher DERS total score and higher scores on Nonacceptance, Goals, Strategies, and Clarity. By contrast, no significant differences in DERS total or subscale scores emerged between participants with and without anxiety disorders, or between participants with and without depressive disorders. Current substance use disorder was associated with higher DERS total score and higher scores on Nonacceptance, Goals, Strategies, Clarity, and Awareness. No significant group differences were observed for the Impulse subscale in either psychiatric comorbidity or current substance use disorder comparisons.

### 3.3. Correlations Between DERS Scores and Continuous Clinical Variables

Pearson correlation analyses between DERS scores and continuous clinical variables are reported in Table 4. DERS total score was not significantly associated with age. Significant positive correlations were observed between DERS total score and ASRS, WURS, ZUNG, STAI-State, STAI-Trait, BIS, IAT total, and CAT-Q total, whereas a significant negative correlation was observed with AAQoL. The strongest correlations were observed with STAI-Trait, WURS, STAI-State, ZUNG, and AAQoL. At the subscale level, Nonacceptance, Goals, Strategies, Clarity, and Awareness showed multiple significant associations, whereas the Impulse subscale showed fewer and weaker correlations.

### 3.4. Multiple Linear Regression Analyses

A simultaneous-entry multiple linear regression model was conducted with DERS total score as the dependent variable. Variables significantly associated with DERS total score in univariate analyses were entered as independent variables. The model included ADHD presentation, psychiatric comorbidity, current substance use disorder (SUD), ASRS, WURS, ZUNG, STAI-State, STAI-Trait, AAQoL, BIS, and IAT total. CAT-Q was not included due to the smaller number of available observations. Results are presented in Table 5.

The model accounted for 40.7% of the variance in DERS total score (adjusted R^2^ = 0.363). In the simultaneous-entry model, current SUD, ASRS, WURS, STAI-Trait, and AAQoL were significant predictors. The remaining variables were not significant. A stepwise regression analysis was subsequently conducted. The final model retained AAQoL, WURS, STAI-Trait, current SUD, and ASRS. This model was statistically significant, F(5, 153) = 19.58, *p* < 0.001, explaining 39.0% of the variance in DERS total score (adjusted R^2^ = 0.370). Results of the final model are reported in Table 6. Current SUD was coded as 1 = present and 2 = absent; therefore, the negative coefficient indicates lower DERS scores in participants without current SUD compared with those with current SUD.

## 4. Discussion

### 4.1. Principal Findings and DERS Profile

The present cross-sectional study characterized emotional dysregulation (ED) in 231 adults with ADHD using the Difficulties in Emotion Regulation Scale (DERS), with the aim of examining its multidimensional structure and clinical correlates.

Overall, adults with ADHD showed elevated DERS total scores relative to reference data [37,38]. When subscale scores were interpreted against the Italian reference values, the most pronounced elevation emerged for emotional clarity, followed by difficulties in goal-directed behavior under distress, nonacceptance of emotional responses, and impulse-control difficulties. In contrast, Strategies showed a more modest elevation, and Awareness was only slightly higher than the Italian reference value. This pattern suggests that the emotional dysregulation profile in this sample was characterized primarily by difficulties in understanding emotional states, maintaining goal-directed behavior under distress, accepting emotional responses, and controlling impulses when emotionally activated.

This profile is consistent with disrupted prefrontal–limbic connectivity and altered dynamic network interactions described in the neurobiological literature on ADHD [43,44], and supports the conceptualization of emotional dysregulation as a multidimensional dimension of the disorder rather than a unitary or secondary symptom.

The prominence of emotional clarity deficits aligns with evidence that ADHD is associated with aberrant processing in brain regions critical for emotional awareness, including the insula and anterior cingulate cortex [43,44]. Persistent attentional difficulties may further impede the sustained monitoring of internal emotional signals, while chronic exposure to poorly understood emotions may hinder the development of coherent emotional schemas. Impaired goal-directed behavior during distress reflects the intersection of emotional and executive dysfunction characteristic of ADHD [13], with individuals struggling to maintain goal-oriented focus or resist avoidance when experiencing negative affect [45]. Although Strategies scores were elevated relative to reference values, this elevation was smaller than that observed for several other DERS dimensions. Therefore, difficulties in perceived access to emotion regulation strategies should be interpreted as part of the broader dysregulation profile, rather than as one of the most pronounced alterations in this sample [46].

Although Awareness was only slightly elevated relative to Italian reference values, its role remains clinically relevant because emotional awareness represents a foundational component of broader emotion-regulation processes [47,48].

### 4.2. Clinical Subgroup Differences

ADHD presentation was significantly associated with the severity of emotional dysregulation. Individuals with the combined presentation showed greater emotional dysregulation than those with the predominantly inattentive presentation, suggesting that hyperactive-impulsive symptoms may contribute to emotional difficulties beyond the effects of inattention alone. This finding is consistent with Barkley’s model of behavioral inhibition as a foundational executive function [49]: impaired inhibitory control may both directly interfere with the suppression of emotionally driven impulses and reduce the capacity to engage in deliberate, top-down regulatory strategies [50]. These results support an integrated affective-executive framework that recognizes emotional dysregulation as a clinically relevant dimension of ADHD’s clinical complexity [51,52]. It should be noted, however, that the hyperactive-impulsive subgroup comprised only four individuals, severely limiting statistical power; the significant subtype effect is therefore driven entirely by the combined vs. inattentive contrast, and future studies with larger hyperactive-impulsive subsamples are needed.

Psychiatric comorbidity was significantly associated with higher emotional dysregulation. Individuals with concomitant psychiatric conditions showed higher DERS total scores and higher scores on Nonacceptance, Goals, Strategies, and Clarity compared with those without psychiatric comorbidity, supporting transdiagnostic models in which emotional dysregulation may function as a shared vulnerability factor across disorders rather than as a secondary consequence of ADHD alone [53,54,55]. When anxiety and depressive disorders were examined separately, neither diagnostic subgroup showed significant differences in DERS total or subscale scores. This suggests that the association between overall psychiatric comorbidity and emotional dysregulation may reflect broader clinical complexity rather than the effect of anxiety or depressive disorders alone [56,57]. These associations are correlational in nature; the cross-sectional design does not permit causal inference, and the directionality of the relationships between comorbidity and regulatory difficulties cannot be determined from these data.

### 4.3. Impulsivity, Substance Use, Internet Use, and Camouflaging

Impulsivity (BIS-11) and emotional dysregulation (DERS) showed only a modest association, challenging the view that emotional difficulties are secondary manifestations of impulsivity and instead suggesting that emotional dysregulation encompasses broader deficits across emotional clarity, awareness, and acceptance. Neurobiologically, this dissociation is mirrored by diverging neural pathways: impulsivity is primarily linked to ventromedial and orbitofrontal regions, whereas ED also recruits limbic structures and their prefrontal connections [58]. From a clinical perspective, these findings indicate that while stimulant medications may effectively reduce impulsivity, adjunctive psychotherapy is often necessary to specifically address emotion regulation deficits [59].

Current substance use disorder (SUD) was significantly associated with elevated emotional dysregulation, with higher scores on DERS total, Nonacceptance, Goals, Strategies, Clarity, and Awareness. This pattern is consistent with models linking substance use to difficulties in affect regulation and maladaptive coping [21,60,61]. Notably, DERS Impulse subscale scores did not differ significantly between individuals with and without SUD, suggesting that substance use in this population may be driven less by trait impulsivity and more by the need to regulate intense emotional distress [62], consistent with self-medication and negative reinforcement models of addiction [63].

In the multiple regression model, current SUD remained independently associated with DERS total score after accounting for ADHD presentation, psychiatric comorbidity, ADHD symptom measures, depressive and anxiety symptoms, quality of life, impulsivity, and problematic internet use. From a clinical perspective, treatment of comorbid ADHD and SUD should directly address underlying emotional vulnerability; interventions such as Dialectical Behavior Therapy (DBT) may be particularly beneficial in developing functional alternatives to substance use [64].

Problematic internet use (IAT) showed a modest positive correlation with DERS total score and with selected DERS subscales. However, IAT was not retained as an independent predictor in the multivariate models, suggesting that its association with emotional dysregulation may overlap with broader symptom burden, trait anxiety, substance use, or ADHD-related quality of life. The relationship is likely bidirectional: individuals with greater regulatory difficulties may increasingly rely on online activities for distraction or emotional avoidance, while excessive internet use may further impair emotional functioning through sleep disruption, social withdrawal, and reduced engagement in adaptive coping [65]. These findings highlight the importance of systematically assessing digital behaviors in adults with ADHD and integrating behavioral strategies targeting problematic internet use with emotion regulation interventions.

The Strategies subscale was associated with some clinical variables, including ADHD presentation, psychiatric comorbidity, current SUD, ADHD symptom measures, depressive symptoms, anxiety symptoms, ADHD-related quality of life, impulsivity, and problematic internet use. However, its elevation relative to Italian reference values was smaller than that observed for Clarity, Goals, Nonacceptance, and Impulse. Therefore, difficulties in perceived access to emotion regulation strategies should be interpreted as one component of the broader emotional dysregulation profile rather than as the central dimension of impairment in this sample [66].

### 4.4. Clinical Implications

Academic difficulties were highly prevalent in the sample and remain an important clinical context for interpreting functional impairment in adults with ADHD. However, the present analyses do not establish academic discontinuation as an independent correlate of emotional dysregulation. Future longitudinal studies should specifically examine whether emotional dysregulation contributes to academic interruption and whether academic failure, in turn, exacerbates emotional dysregulation through reduced self-efficacy and increased stress [67,68,69,70].

These findings highlight the importance of routinely incorporating multidimensional assessments of emotional dysregulation into the clinical evaluation of adults with ADHD, particularly in individuals presenting with psychiatric comorbidities, current substance use disorder, greater ADHD symptom burden, trait anxiety, poorer ADHD-related quality of life, or problematic internet use. Reliance on aggregate scores alone may obscure clinically meaningful individual profiles; a dimensional approach is therefore essential for more precise and individualized treatment planning.

Comprehensive clinical management should integrate the treatment of core ADHD symptoms with targeted interventions addressing emotion regulation. Skills-based approaches—such as DBT-informed strategies, emotion regulation therapy, mindfulness-based interventions, and Acceptance and Commitment Therapy (ACT)—appear especially well suited for this population, particularly when adapted to account for executive dysfunction [19,71]. Because difficulties in perceived access to emotion regulation strategies were present but not among the most pronounced elevations relative to reference values, interventions targeting regulation strategies should be considered as part of a broader approach addressing multiple DERS dimensions, including clarity, goal-directed behavior under distress, nonacceptance, and impulse control.

From a pragmatic clinical perspective, these findings have direct implications for treatment sequencing in adult outpatient settings. Pharmacological treatment targeting core ADHD symptoms (e.g., stimulants or atomoxetine) may represent a necessary first step to enhance attentional control and reduce cognitive overload [10,16], thereby creating conditions for more effective engagement in psychotherapy. However, pharmacotherapy alone is unlikely to adequately address emotional dysregulation. In cases characterized by severe emotional dysregulation, substance use, or maladaptive coping behaviors, early integration of structured psychotherapeutic interventions—particularly those focused on emotion regulation (e.g., DBT-informed approaches)—may be indicated from the outset rather than deferred. The presence of psychiatric comorbidity may further require prioritizing stabilization of acute affective or anxiety symptoms alongside ADHD-specific treatment. Overall, these results support a flexible, patient-tailored sequencing model in which the relative prominence of emotional dysregulation and comorbidity guides the timing and integration of pharmacological and psychological interventions.

### 4.5. Limitations, Strengths, and Future Directions

The cross-sectional design of this study precludes causal inferences and does not elucidate the temporal relationships between emotional dysregulation, psychiatric comorbidity, substance use, and functional impairment. As all constructs were evaluated through self-report measures, results may be subject to shared method variance and potential reporting or insight-related biases. The single-center nature of the study and the relatively young age of the cohort may constrain generalizability, and some subgroup analyses may have been underpowered. The performance of multiple statistical comparisons increases the risk of Type I errors, and the multiple regression model included a relatively large number of predictors, raising the risk of overfitting; findings should therefore be interpreted with caution and require replication in independent samples.

A notable limitation concerns the relatively low contribution of impulse control difficulties within the sample. Although impulsivity is a core feature of ADHD, the Impulse subscale of the DERS showed comparatively weaker and less consistent associations with clinical variables relative to other DERS dimensions. Several explanations may account for this pattern. First, a floor effect may have limited variance in this subscale within a clinically selected ADHD sample.

Second, limited variance in this subscale may also reflect the clinical and treatment characteristics of the sample. Third, there may be a genuine dissociation between impulsive behavior as a behavioral trait and the broader construct of emotional dysregulation as measured by self-report. Finally, the conceptual overlap between the DERS-Impulse subscale and the BIS-11 may have introduced redundancy and attenuated unique variance. This finding limits conclusions regarding the role of impulse control within the overall ED profile and should be addressed in future studies using behavioral or neuropsychological measures of impulsivity. Current pharmacological and psychotherapeutic treatment represents an additional limitation. Many participants were receiving ADHD pharmacotherapy and/or psychotherapy at the time of assessment, and ongoing treatment may have influenced not only impulsivity-related outcomes but also DERS total and subscale scores, ADHD symptom measures, group differences, and associations with clinical and functional variables. Although sensitivity analyses were conducted to examine whether findings differed when controlling for current ADHD medication status, residual confounding related to treatment type, dosage, duration, adherence, treatment response, and psychotherapy exposure cannot be excluded.

An additional limitation concerns the clinical heterogeneity of psychiatric comorbidity. Although overall psychiatric comorbidity was associated with higher DERS scores, anxiety and depressive disorders considered separately were not significantly associated with DERS total or subscale scores. These subgroup analyses should be interpreted cautiously because diagnostic categories were not mutually exclusive, some subgroups were relatively small, and clinical state at the time of assessment was not systematically controlled. Future studies should examine specific psychiatric comorbidity profiles in larger samples and assess symptom state and severity at the time of evaluation.

The hyperactive-impulsive ADHD subgroup was significantly underrepresented (n = 4), precluding meaningful subtype comparisons beyond the combined vs. inattentive contrast. Specific neurodevelopmental comorbidity profiles, including autism spectrum disorder, were not examined in the present Results section and should be addressed in future studies with larger subsamples. The absence of a non-ADHD control group constitutes a notable limitation and precludes definitive conclusions regarding ADHD-specific effects, limiting the ability to disentangle ADHD-related dysregulation from that associated with comorbid conditions or general clinical complexity. Finally, the exclusion of patients with personality disorders—particularly borderline personality disorder (BPD), which is characterized by marked emotional dysregulation—limits the generalizability of findings to more complex and clinically representative populations in which ADHD-BPD comorbidity is relatively common. Future studies should include comorbid samples to better disentangle disorder-specific and shared mechanisms within a transdiagnostic framework.

Strengths of the present study include a large, clinically well-characterized adult ADHD sample, structured diagnostic assessment (DIVA 5), multidimensional evaluation of emotional dysregulation, and examination of clinically relevant variables such as substance use and internet addiction within a real-world clinical setting.

Longitudinal research is needed to clarify temporal and causal relationships between emotional dysregulation and clinical outcomes. Randomized trials should test emotion-focused interventions adapted for ADHD and evaluate whether improvements in emotional regulation mediate functional outcomes. Mechanistic studies integrating neurobiological, cognitive, and ecological momentary approaches would further clarify the substrates of emotional dysregulation. Research on digital behaviors, camouflaging, and precision-medicine approaches may help identify subgroups with distinct regulatory profiles and optimize personalized interventions. Studies including matched non-ADHD clinical and healthy control groups are needed to establish the specificity of the emotional dysregulation profiles identified in this sample.

## 5. Conclusions

This study shows that emotional dysregulation is commonly observed in adults with ADHD and is associated with clinically relevant indicators, including ADHD symptom burden, trait anxiety, current SUD, and ADHD-related quality of life. Compared with reference values, the most pronounced DERS subscale elevation was observed for Clarity, followed by Goals, Nonacceptance, and Impulse, whereas Strategies showed a more modest elevation and Awareness was only slightly higher than the Italian reference value.

Impulsivity showed a modest bivariate association with DERS total score but was not an independent predictor in the multivariate model, suggesting that emotional dysregulation is not fully captured by impulsivity alone. In the final regression model, current SUD, current ADHD symptoms, retrospective childhood ADHD symptoms, trait anxiety, and ADHD-related quality of life were independently associated with DERS total score.

Because of the cross-sectional design and the absence of non-ADHD clinical and healthy control groups, these findings do not establish whether emotional dysregulation represents a definitional domain of ADHD, an associated feature, or a marker of a specific clinical subtype. Longitudinal and controlled studies are needed to clarify the specificity, temporal direction, and clinical implications of emotional dysregulation in adults with ADHD.

## Figures and Tables

**Table 2:** Descriptive statistics of DERS scores in the current ADHD sample and reference values from validation studies.

DERS	Current ADHD Sample N	Current ADHD Sample M (SD)	Min–Max	Italian Reference Sample M (SD)	Original Validation Sample M (SD), Women/Men
DERS total	212	115.02 (23.99)	48–178	81.10 (18.50)	77.99 (20.72)/80.66 (18.79)
Nonacceptance	211	18.38 (6.38)	2–30	12.40 (4.50)	11.65 (4.72)/11.55 (4.20)
Goals	212	20.60 (3.67)	8–28	14.60 (4.40)	14.41 (4.95)/14.34 (5.16)
Strategies	210	20.10 (7.07)	3–38	17.10 (6.20)	16.16 (6.19)/16.23 (6.26)
Impulse	210	16.86 (4.95)	6–31	12.10 (4.30)	10.82 (4.41)/11.55 (4.59)
Clarity	210	22.77 (8.69)	4–40	10.40 (3.30)	10.61 (3.80)/10.74 (3.67)
Awareness	212	16.14 (5.55)	4–26	14.50 (4.00)	14.34 (4.60)/16.26 (4.61)

Note. DERS = Difficulties in Emotion Regulation Scale; ADHD = attention-deficit/hyperactivity disorder. Higher scores indicate greater difficulties in emotion regulation. Italian reference values are based on an Italian validation sample of 323 students [37]. Original validation values reported descriptive statistics separately for women and men [8]. Sample sizes in the current ADHD sample vary slightly across DERSs due to missing data. These values should be interpreted as reference values from validation samples rather than population norms.

**Table 3:** Group comparisons of DERS total and subscale scores across binary socio-demographic and clinical variables.

Grouping Variable	DERS	Group 1 n	Group 1 M (SD)	Group 2 n	Group 2 M (SD)	t(df)	*p*	Cohen’s d
Sex: male vs. female	DERS total	122	113.98 (21.83)	89	116.54 (26.85)	−0.74 (165.38)	0.462	−0.11
	Nonacceptance	121	17.98 (5.89)	89	18.97 (7.01)	−1.10 (208)	0.272	−0.15
	Goals	122	20.33 (3.77)	89	21.00 (3.55)	−1.31 (209)	0.191	−0.18
	Strategies	120	19.83 (7.24)	89	20.40 (6.90)	−0.58 (207)	0.565	−0.08
	Impulse	120	16.90 (4.83)	89	16.75 (5.14)	0.21 (207)	0.832	0.03
	Clarity	120	22.89 (7.92)	89	22.69 (9.69)	0.16 (166.50)	0.870	0.02
	Awareness	122	15.72 (5.58)	89	16.78 (5.49)	−1.37 (209)	0.174	−0.19
ADHD presentation: combined vs. inattentive	DERS total	161	117.39 (24.53)	44	104.50 (19.73)	3.21 (203)	0.002	0.55
	Nonacceptance	160	18.88 (6.38)	44	15.50 (5.41)	3.21 (202)	0.002	0.55
	Goals	161	20.82 (3.71)	44	19.66 (3.51)	1.86 (203)	0.064	0.32
	Strategies	159	21.18 (6.99)	44	16.25 (6.42)	4.21 (201)	<0.001	0.72
	Impulse	159	16.89 (5.13)	44	16.55 (4.47)	0.40 (201)	0.689	0.07
	Clarity	159	23.21 (8.85)	44	20.89 (8.10)	1.57 (201)	0.118	0.27
	Awareness	161	16.48 (5.89)	44	14.66 (4.00)	2.40 (99.74)	0.018	0.33
Psychiatric comorbidity: present vs. absent	DERS total	160	118.14 (21.88)	52	105.40 (27.66)	3.03 (72.89)	0.003	0.54
	Nonacceptance	159	19.08 (5.82)	52	16.25 (7.53)	2.48 (72.04)	0.016	0.45
	Goals	160	20.93 (3.54)	52	19.62 (3.92)	2.25 (210)	0.025	0.36
	Strategies	158	20.73 (6.81)	52	18.17 (7.56)	2.28 (208)	0.023	0.37
	Impulse	158	17.18 (4.92)	52	15.90 (4.97)	1.61 (208)	0.108	0.26
	Clarity	158	23.70 (8.56)	52	19.94 (8.55)	2.75 (208)	0.006	0.44
	Awareness	160	16.34 (5.71)	52	15.52 (5.04)	0.93 (210)	0.353	0.15
Anxiety disorder: present vs. absent	DERS total	59	113.20 (20.63)	153	115.72 (25.20)	−0.75 (127.78)	0.457	−0.11
	Nonacceptance	59	18.51 (5.81)	152	18.33 (6.61)	0.18 (209)	0.855	0.03
	Goals	59	20.66 (3.11)	153	20.58 (3.88)	0.14 (210)	0.888	0.02
	Strategies	58	20.02 (7.31)	152	20.13 (7.00)	−0.10 (208)	0.922	−0.02
	Impulse	58	16.03 (5.06)	152	17.18 (4.89)	−1.50 (208)	0.135	−0.23
	Clarity	58	21.71 (8.44)	152	23.18 (8.77)	−1.10 (208)	0.274	−0.17
	Awareness	59	15.63 (5.59)	153	16.34 (5.54)	−0.84 (210)	0.403	−0.13
Depressive disorder: present vs. absent	DERS total	35	119.23 (24.31)	177	114.19 (23.91)	1.14 (210)	0.257	0.21
	Nonacceptance	35	18.57 (6.09)	176	18.34 (6.46)	0.20 (209)	0.846	0.04
	Goals	35	21.00 (3.44)	177	20.53 (3.72)	0.70 (210)	0.486	0.13
	Strategies	35	20.51 (5.89)	175	20.01 (7.29)	0.38 (208)	0.702	0.07
	Impulse	35	17.06 (3.97)	175	16.82 (5.14)	0.26 (208)	0.799	0.05
	Clarity	35	24.83 (8.35)	175	22.36 (8.72)	1.54 (208)	0.125	0.29
	Awareness	35	17.26 (5.60)	177	15.92 (5.53)	1.30 (210)	0.194	0.24
Current SUD: present vs. absent	DERS total	52	125.67 (19.26)	159	111.30 (24.26)	4.37 (108.28)	<0.001	0.62
	Nonacceptance	52	21.19 (5.19)	158	17.38 (6.43)	3.88 (208)	<0.001	0.62
	Goals	52	21.58 (3.07)	159	20.26 (3.80)	2.27 (209)	0.024	0.36
	Strategies	52	22.87 (6.87)	157	19.14 (6.91)	3.37 (207)	0.001	0.54
	Impulse	52	17.48 (4.50)	157	16.65 (5.11)	1.05 (207)	0.297	0.17
	Clarity	52	24.98 (8.38)	157	21.97 (8.66)	2.19 (207)	0.030	0.35
	Awareness	52	17.73 (5.16)	159	15.60 (5.60)	2.43 (209)	0.016	0.39

Note. DERS = Difficulties in Emotion Regulation Scale; ADHD = attention-deficit/hyperactivity disorder; SUD = substance use disorder. Independent-samples *t*-tests were used to compare DERS total and subscale scores across binary groups. Welch’s correction was applied when Levene’s test indicated unequal variances. Cohen’s d was computed using the pooled standard deviation. For sex, Group 1 = male and Group 2 = female. For ADHD presentation, Group 1 = combined and Group 2 = inattentive; participants with a hyperactive/impulsive presentation were excluded from this comparison because of the very small subgroup size (n = 4). For psychiatric comorbidity, anxiety disorder, depressive disorder, and current SUD, Group 1 = presence and Group 2 = absence of the condition. Positive Cohen’s d values indicate higher scores in Group 1; negative values indicate higher scores in Group 2. Anxiety and depressive disorder groups were not mutually exclusive, as participants could present with more than one psychiatric comorbidity. Sample sizes vary slightly across analyses due to missing data.

**Table 4:** Pearson correlations between DERS scores and continuous clinical variables.

Variable	DERS Total	Nonacceptance	Goals	Strategies	Impulse	Clarity	Awareness
Age	0.039	0.059	0.032	0.045	0.033	−0.012	−0.046
ASRS	0.273 **	0.209 **	0.138 *	0.281 **	0.157 *	0.151 *	0.139 *
WURS	0.396 **	0.388 **	0.164 *	0.209 **	0.105	0.323 **	0.240 **
ZUNG	0.381 **	0.285 **	0.244 **	0.314 **	0.182 *	0.214 **	0.278 **
STAI-State	0.385 **	0.310 **	0.252 **	0.199 **	0.170 *	0.354 **	0.182 *
STAI-Trait	0.423 **	0.288 **	0.291 **	0.287 **	0.224 **	0.304 **	0.265 **
AAQoL	−0.358 **	−0.337 **	−0.202 **	−0.248 **	−0.022	−0.264 **	−0.233 **
BIS	0.223 **	0.069	0.207 **	0.195 **	0.107	0.157 *	0.121
IAT total	0.208 **	0.081	0.218 **	0.186 **	0.133	0.180 *	0.036
CAT-Q total	0.255 **	0.216 *	0.146	0.175	0.094	0.148	0.204 *

Note. Values are Pearson correlation coefficients. DERS = Difficulties in Emotion Regulation Scale; ASRS = Adult ADHD Self-Report Scale; WURS = Wender Utah Rating Scale; STAI = State-Trait Anxiety Inventory; AAQoL = Adult ADHD Quality of Life Scale; BIS = Barratt Impulsiveness Scale; IAT = Internet Addiction Test; CAT-Q = Camouflaging Autistic Traits Questionnaire. Higher AAQoL scores indicate better quality of life. Sample sizes varied across correlations due to missing data. * *p* < 0.05; ** *p* < 0.01.

**Table 5:** Multiple linear regression model for DERS total score.

Predictor	*B*	*SE*	*β*	*t*	*p*
ADHD presentation	−2.06	4.34	−0.033	−0.48	0.635
Psychiatric comorbidity	−2.18	4.11	−0.037	−0.53	0.597
Current SUD	−10.15	3.95	−0.173	−2.57	0.011
ASRS	1.41	0.57	0.166	2.50	0.013
WURS	0.39	0.12	0.247	3.44	0.001
ZUNG	−0.02	0.19	−0.008	−0.09	0.929
STAI-State	−0.13	0.20	−0.067	−0.68	0.499
STAI-Trait	0.76	0.24	0.313	3.21	0.002
AAQoL	−0.40	0.11	−0.245	−3.61	<0.001
BIS	−0.01	0.11	−0.009	−0.12	0.907
IAT total	0.21	0.12	0.128	1.78	0.077

Model fit: *F*(11, 147) = 9.19, *p* < 0.001, *R*^2^ = 0.407, adjusted *R*^2^ = 0.363. Note. DERS = Difficulties in Emotion Regulation Scale; ADHD = attention-deficit/hyperactivity disorder; SUD = substance use disorder; ASRS = Adult ADHD Self-Report Scale; WURS = Wender Utah Rating Scale; ZUNG = Zung Self-Rating Depression Scale; STAI = State-Trait Anxiety Inventory; AAQoL = Adult ADHD Quality of Life Scale; BIS = Barratt Impulsiveness Scale; IAT = Internet Addiction Test. Predictors were entered simultaneously using the enter method. Variables included in the model were those significantly associated with DERS total score in univariate analyses. ADHD presentation was coded as 1 = combined and 2 = inattentive; psychiatric comorbidity and current SUD were coded as 1 = present and 2 = absent. Therefore, negative coefficients for these binary variables indicate lower DERS scores in the group coded 2 relative to the group coded 1. CAT-Q was not included in the main model because of the substantially smaller number of available observations. The model was based on complete cases for all included predictors.

**Table 6:** Stepwise multiple linear regression model for DERS total score.

Predictor Retained in Final Model	*B*	*SE*	*β*	*t*	*p*
AAQoL	−0.41	0.11	−0.256	−3.89	<0.001
WURS	0.37	0.10	0.231	3.54	0.001
STAI-Trait	0.67	0.16	0.276	4.19	<0.001
Current SUD	−11.79	3.80	−0.201	−3.10	0.002
ASRS	1.61	0.55	0.189	2.93	0.004

Final model fit: *F*(5, 153) = 19.58, *p* < 0.001, *R*^2^ = 0.390, adjusted *R*^2^ = 0.370. Note. DERS = Difficulties in Emotion Regulation Scale; AAQoL = Adult ADHD Quality of Life Scale; WURS = Wender Utah Rating Scale; STAI-Trait = Trait scale of the State-Trait Anxiety Inventory; SUD = substance use disorder; ASRS = Adult ADHD Self-Report Scale. Stepwise selection was conducted using variables significantly associated with DERS total score in univariate analyses. Current SUD was coded as 1 = present and 2 = absent; therefore, the negative coefficient indicates lower DERS scores among participants without current SUD compared with those with current SUD. Higher AAQoL scores indicate better quality of life. The model was based on complete cases for all included predictors.

## Data Availability

The datasets generated and analyzed during the current study are not publicly available due to privacy and ethical restrictions related to the clinical nature of the data but are available from the corresponding author upon reasonable request.

## Supplementary Materials

The following supporting information can be downloaded at: https://www.mdpi.com/article/10.3390/brainsci16060577/s1, Figure S1: Trends of DERS Total Score.
