# Supplementary figures and images for: Emotional Dysregulation as a Clinically Relevant Dimension of Adult ADHD: A Multidimensional Clinical Study
Source: Brain Sci. 2026 May 29;16(6):577. doi: 10.3390/brainsci16060577 (PMC13297159; doi:10.3390/brainsci16060577)

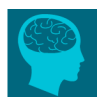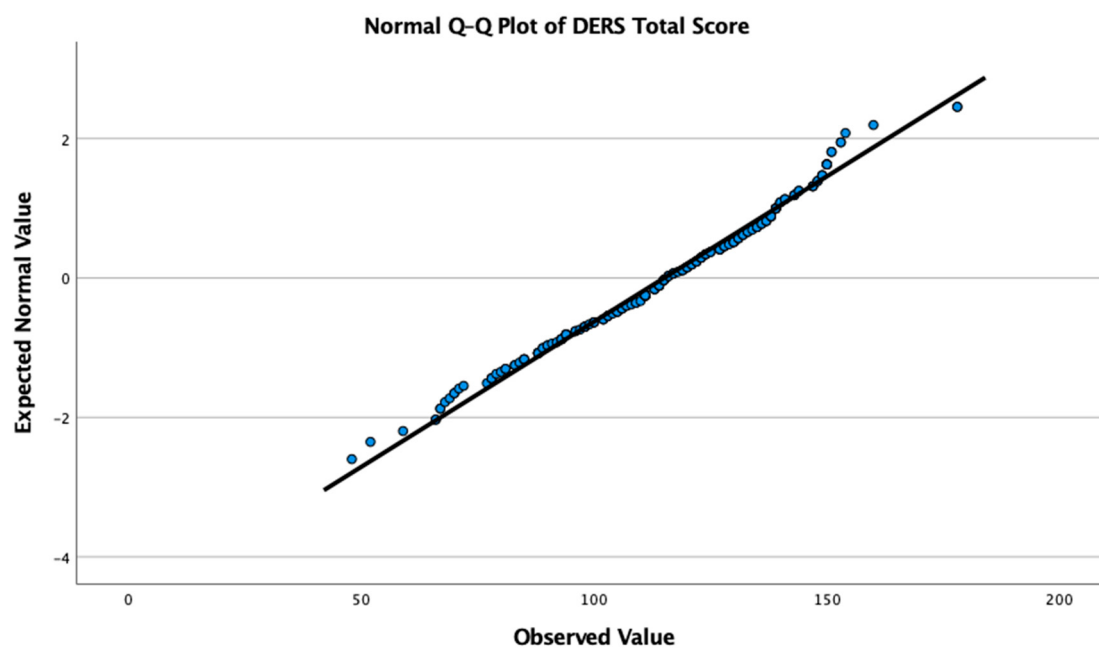

Figure S1. Trends of DERS Total Score.

Supplement: Supplementary file 1 [file brainsci-16-00577-s001.zip › brainsci-4250673-supplementary.pdf]
